# Development of ^111^In-Labeled Monoclonal Antibodies Targeting SFTSV Structural Proteins for Molecular Imaging of SFTS Infectious Diseases by SPECT

**DOI:** 10.3390/molecules30010038

**Published:** 2024-12-26

**Authors:** Takeshi Fuchigami, Mya Myat Ngwe Tun, Yusuke Tanahara, Kodai Nishi, Sakura Yoshida, Kazuma Ogawa, Morio Nakayama, Daisuke Hayasaka

**Affiliations:** 1 Laboratory of Clinical Analytical Sciences, Graduate School of Medical Sciences, Kanazawa University, Kakuma-machi, Kanazawa 920-1192, Japan; kogawa@p.kanazawa-u.ac.jp; 2 Center for Vaccines and Therapeutic Antibodies for Emerging Infectious Diseases, Shimane University, Izumo 690-8504, Japan; ntmyamyat@med.shimane-u.ac.jp; 3 Department of Virology, Department of Tropical Viral Vaccine Development, Institute of Tropical Medicine, Nagasaki University, 1-12-4 Sakamoto, Nagasaki 852-8523, Japan; 4 Department of Hygienic Chemistry, Graduate School of Biomedical Sciences, Nagasaki University, 1-14 Bunkyo-machi, Nagasaki 852-8521, Japan; tanahara.yusuke@gmail.com (Y.T.); yoshida-s@nagasaki-u.ac.jp (S.Y.); nakmorio@gmail.com (M.N.); 5 Department of Radioisotope Medicine, Atomic Bomb Disease Institute, Nagasaki University, 1-12-4 Sakamoto, Nagasaki 852-8523, Japan; koudai@nagasaki-u.ac.jp; 6 Institute for Frontier Science Initiative, Kanazawa University, Kakuma-machi, Kanazawa 920-1192, Japan; 7 Laboratory of Veterinary Microbiology, Joint Graduate School of Veterinary Medicine, Yamaguchi University, 1677-1 Yoshida, Yamaguchi 753-8511, Japan

**Keywords:** severe fever thrombocytopenia syndrome, single-photon emission computed tomography, indium-111, antibody

## Abstract

No effective vaccines or treatments are currently available for severe fever with thrombocytopenia syndrome (SFTS), a fatal tick-borne infectious disease caused by the SFTS virus (SFTSV). This study evaluated the potential of ^111^In-labeled anti-SFTSV antibodies targeting SFTSV structural proteins as single-photon emission computed tomography (SPECT) imaging agents for the selective visualization of SFTSV-infected sites. This study used nuclear medicine imaging to elucidate the pathology of SFTS and assess its therapeutic efficacy. Immunostaining experiments confirmed that the anti-SFTSV antibody (N-mAb), which targets the N protein, specifically accumulated in SFTSV-infected Vero E6 cells. ^111^In-labeled N-mAb was successfully prepared using a diethylenetriaminepentaacetic acid (DTPA) chelator, resulting in [^111^In]In-DTPA-N-mAb with high radiochemical purity exceeding 95% and a radiochemical yield of 55%. Cell-binding assays using SFTSV-infected Vero E6 cells demonstrated that [^111^In]In-DTPA-N-mAb binding was detectable even without membrane permeabilization, with the binding intensity correlating with infection levels. In vivo studies using SFTSV-infected A129 mice showed high spleen accumulation of [^111^In]In-DTPA-N-mAb (87.5% ID/g), consistent with SFTSV tropism, compared to 12.3% ID/g in mock-infected mice. SPECT/CT imaging clearly revealed high radioactivity in these regions. Although nonspecific accumulation was noted in the liver and spleen, this issue may be mitigated through antibody modifications such as fragmentation or PEGylation. Overall, [^111^In]In-DTPA-N-mAb is a promising imaging agent for non-invasive visualization of SFTSV-infected sites and may aid in elucidating SFTS pathology and assessing therapeutic efficacy.

## 1. Introduction

Severe Fever with Thrombocytopenia Syndrome (SFTS) is a lethal infectious disease caused by the SFTS virus (SFTSV), which was officially recognized in China in 2011 [1]. SFTSV belongs to the *Bandavirus* genus within the Phenuiviridae family and is primarily transmitted by ticks [2]. *Haemaphysalis longicornis* (the long-horned tick) is considered the main vector of infection, with infections typically occurring through tick bites [3]. However, recent reports have confirmed that SFTSV can be transmitted between humans through bodily fluids such as semen and respiratory secretions [4,5].

The clinical symptoms of SFTS are non-specific and include fever, leukopenia, thrombocytopenia, and gastrointestinal distress [6]. Neurological symptoms and bleeding tendencies can arise in severe cases, potentially leading to multiple organ failure and death [7,8]. The fatality rate of SFTS is exceptionally high, with early studies reporting rates up to 30% [7,8,9]. Although more recent research has shown a decline in the fatality rate, it still exceeds 5% [10]. Importantly, the fatality rate remains significantly higher in older adults. The progression of SFTS can be divided into three main stages: febrile, multi-organ failure, and recovery, each characterized by distinct features [6,11]. In SFTSV infection, the virus targets B lymphocytes, reducing antibody production and weakening the immune response. A cytokine storm triggers excessive inflammation, damaging blood vessels, promoting platelet aggregation, and leading to thrombocytopenia. This immune dysfunction and inflammatory response are considered the primary causes of the multi-organ failure [12].

Research on the prognosis of SFTS is ongoing, revealing that factors such as aspartate aminotransferase (AST), creatine kinase (CK), creatine kinase-myocardial band (CKMB), lactate dehydrogenase (LDH), and neurological symptoms are associated with mortality risk in the case of this disease [13,14,15]. However, many of these studies were based on data collected at the time of admission and did not utilize dynamic monitoring data, thereby limiting the conclusions drawn. Studies on antiviral drugs such as ribavirin, favipiravir, the calcium channel blocker nifedipine, and the estrogen receptor modulator bazedoxifene acetate (BZA) are ongoing. However, symptomatic treatment remains the primary approach, and no effective vaccines or definitive treatments have been established [16,17,18,19].

By employing nuclear medicine imaging techniques such as positron emission tomography (PET) and single-photon emission computed tomography (SPECT), it is possible to track disease progression over time in the same individual and identify the locations of disease manifestation. PET and SPECT offer superior detection depths, enabling the construction of tomographic images of any part of the body. This is an advantage over fluorescent imaging, which can only detect a few millimeters beneath the body surface. Additionally, while SPECT is somewhat less accurate than PET in terms of quantification, it offers the advantage of versatility, as it does not require a large cyclotron for isotope production [20,21]. This approach not only helps elucidate mechanisms underlying the onset of SFTS, but also helps develop effective treatments and evaluate their therapeutic efficacy. We previously conducted PET/CT imaging of SFTSV-infected mice using two molecular probes: 2-[^18^F]fluoro-2-deoxy-d-glucose ([^18^F]FDG), which macrophages take up via glucose transporters [22], and [^67^Ga]Ga-citrate, which accumulates in inflammatory tissues after binding to transferrin and enters macrophages and neutrophils [23]. Previously, we found that significant accumulation of [^18^F]FDG and [^68^Ga]Ga-citrate in the gastrointestinal tract, a pathological finding characteristic of SFTS, was observed only in infected mice [24,25]. This indicates that nuclear medicine imaging can non-invasively visualize SFTS. However, both radiotracers accumulate in tissues inflamed by various infections, which poses a challenge to their use in the selective pathological analysis of SFTS. Therefore, there is a need to develop molecular probes that enable more specific in vivo imaging of SFTSV infections.

SFTSV is an RNA virus with a negative-sense, single-stranded genome consisting of three segments: S, M, and L. These genome segments are categorized based on their size, with the L (large) segment being 6.4 kb, M (medium) 3.6 kb, and S (small) 2–3 kb. The L segment encodes the L protein (an RNA-dependent RNA polymerase), the M segment encodes the envelope glycoproteins Gn and Gc, and the S segment encodes the nucleoprotein N [26]. The N protein promotes capsid formation and forms a complex with viral RNA and various RNA polymerase proteins known as the ribonucleoprotein (RNP) complex [27]. This RNP complex is transcribed to synthesize mRNA, making the N protein an essential component for viral replication [28,29]. Additionally, in viruses of the same genus as SFTSV, such as Toscana virus and Rift Valley fever virus, the N protein is highly immunogenic and is considered a major target antigen in the analyses of patient serum samples [30,31].

Therefore, proteins produced by SFTSV, such as N, Gn, and Gc, are expected to serve as a basis for the development of SFTSV-specific imaging agents. In this study, we synthesized ¹¹¹In-labeled antibodies targeting the SFTSV-specific proteins and evaluated their potential as SFTSV-specific in vivo imaging agents.

## 2. Results and Discussion

### 2.1. Screening of SFSV-Targeting Antibodies as Scaffolds for SFTSV Imaging Probes

**Figure 1:**
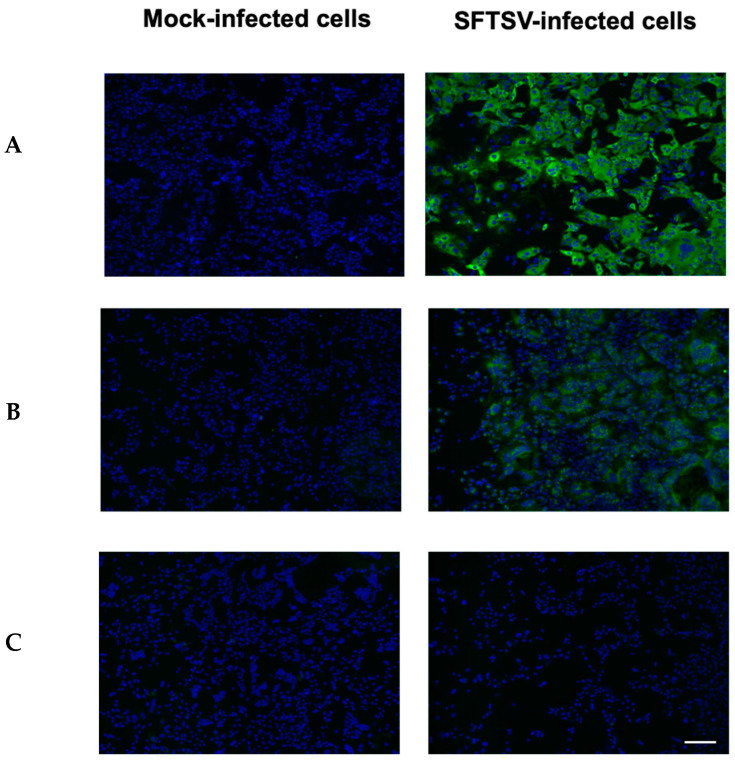
Fluorescence images of the SFTSV antibody targeting N protein (Primary Ab: 4A10) (**A**), Gn protein (2D4) (**B**), and Gc protein (3B4) (**C**) in Vero E6 cells infected with SFTSV at mock-infected or multiplicity of infection (MOI)-0.1. The secondary antibody was FITC-conjugated anti-mouse IgG (green), and DAPI was used for nuclear staining (blue). Scale bar = 100 µm.

To evaluate whether monoclonal antibodies against SFTSV targeting N, Gn, and Gc proteins (N4A10, GN2D4, and GC3B4, respectively) could function as imaging agents, antibody immunostaining was performed on SFTSV-infected Vero E6 cells (Figure 1). The results showed that the anti-SFTSV antibody targeting the N protein (N-mAb) produced no significant fluorescence in non-infected (mock) cells, whereas clear fluorescence was observed in SFTSV-infected cells at multiplicity of infection (MOI)-0.1, indicating effective recognition of the N protein. Additionally, the anti-SFTSV antibody targeting the Gn protein (Gn-mAb) exhibited weak fluorescence in SFTSV-infected cells, whereas no fluorescence was detected for the anti-SFTSV antibody targeting the Gc protein (Gc-mAb). These results suggest that, among the antibodies tested, N-mAb (N4A10) showed the greatest potential as an imaging agent for SFTSV-infected cells. The lack of strong fluorescence with other antibodies may be attributed to factors such as the expression levels and localization of target proteins in infected cells, as well as differences in the antigen recognition abilities of the antibodies. In the case of Ebola virus, which is a negative-sense single-stranded RNA virus with an envelope, the N protein expressed in infected cells is known to change its distribution over time, moving toward the cell membrane, similar to SFTSV [32]. Additionally, as infection could be confirmed by detecting anti-N protein antibodies in the serum of patients with SFTSV infection [31], it is highly likely that the N protein produced within the infected cells is exposed on the cell membrane as the infection progresses, allowing antibodies to access it directly.

### 2.2. Radiosynthesis of ^111^In-Labeled-Diethylenetriaminepentaacetic Acid (DTPA) Antibodies

**Scheme 1:**
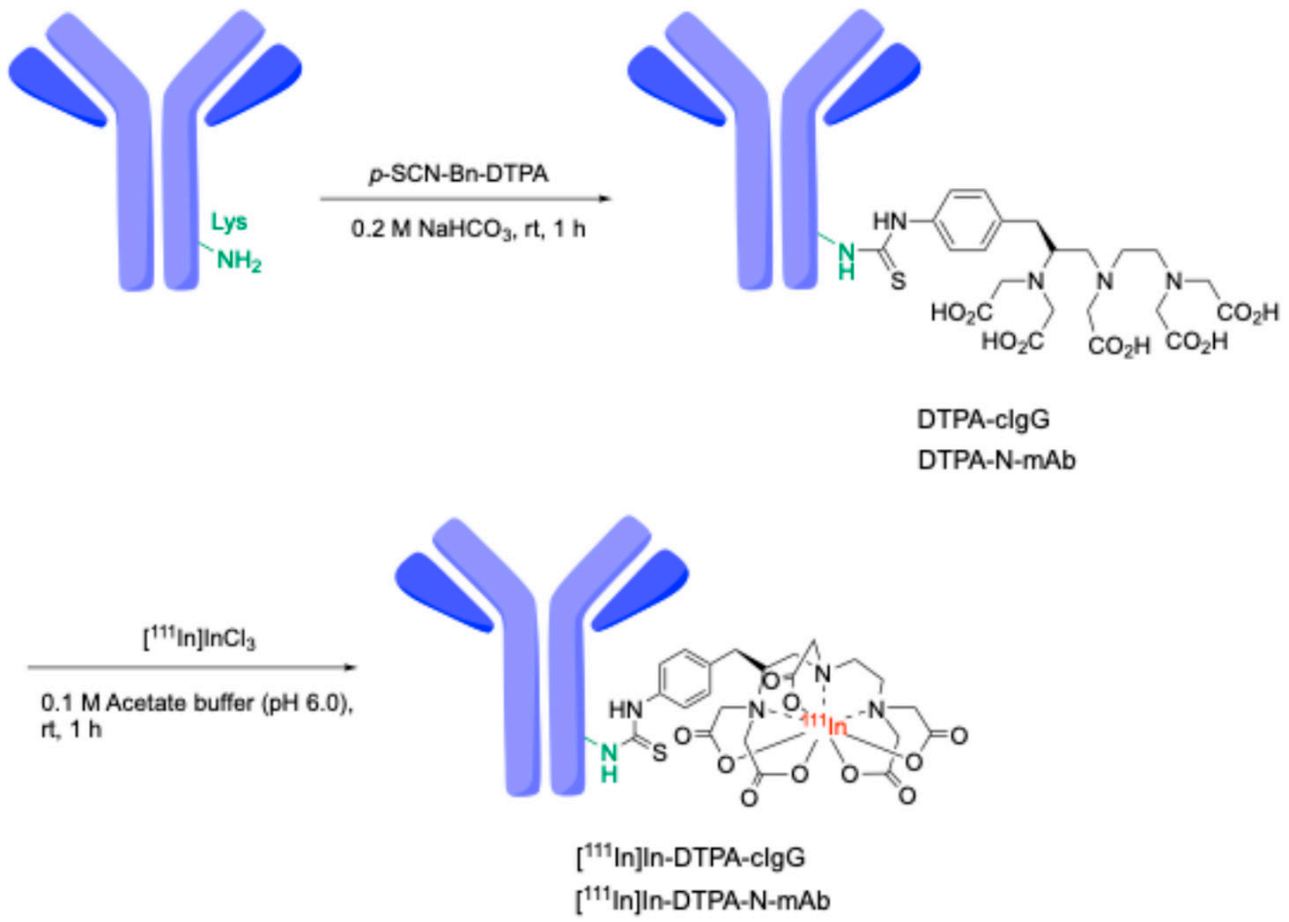
Preparation of [^111^In]In-DTPA-cIgG and [^111^In]In-DTPA-N-mAb.

Cell staining experiments suggest that N-mAb (N4A10) may be useful for in vivo imaging of SFTSV dynamics. However, when using IgG as an imaging probe, its long blood half-life necessitates several days for the clear imaging of tumor tissues. Therefore, we designed a radiotracer labeled with the In-111 radionuclide, which has a half-life of 3.02 days, to facilitate selective SPECT imaging of SFTSV. The synthesis of the precursor antibody and the ^111^In-labeled antibody is shown in Scheme 1. Initially, the N-mAb was conjugated with p-SCN-Bn-DTPA to produce DTPA-N-mAb, which formed a stable chelate with In-111. MALDI-TOF-MS analysis indicated that a DTPA-N-mAb derivative with two molecules of DTPA bound to N-mAb was detected (Figure S1). Subsequently, ^111^In-labeled DTPA-N-mAb ([^111^In]In-DTPA-N-mAb) was prepared using [^111^In]InCl_3_. The resulting radiochemical yield was 55% and the radiochemical purity exceeded 95% determined by instant thin-layer chromatography (iTLC) as shown in Figure S2A. To validate the utility of [^111^In]In-DTPA-N-mAb as an imaging agent for SFTSV, [^111^In]In-DTPA-cIgG derived from an isotype control antibody (cIgG) was used as a negative control. [^111^In]In-DTPA-cIgG was prepared using the same methodology as that for [^111^In]In-DTPA-N-mAb, resulting in a radiochemical yield of 73% and radiochemical purity of over 95% (Figure S2B). It is expected that increasing the amount of DTPA bound to the antibody or using a larger quantity of reactive antibodies would improve the yield, which is particularly important given the high cost of radionuclides in radiopharmacy. However, factors such as the effects on affinity and self-blocking must also be considered. For future clinical applications, where cost efficiency and higher yields are critical, further investigation into yield improvement will be necessary.

### 2.3. Cell Binding Assays of ^111^In-Antibodies to SFTSV-Infected Vero E6 Cells

**Figure 2:**
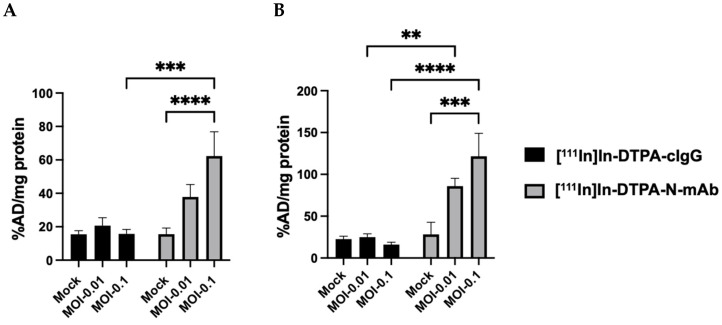
In vitro binding of ^111^In-labeled IgG ([^111^In]In-DTPA-cIgG, [^111^In]In-DTPA-N-mAb) in SFTSV-infected (MOI-0.01, MOI-0.1) and mock-infected Vero E6 cells. Vero E6 cells were processed as only fixation (**A**) or fixation and permeabilization (**B**). ** *p* < 0.01, *** *p* < 0.001, **** *p* < 0.0001 (two-way ANOVA with Tukey’s post hoc test). Values are mean ± SEM, n = 5–10.

Next, we examined the ability of [^111^In]In-DTPA-N-mAb or [^111^In]In-DTPA-cIgG to bind the N protein on the cell membrane surface and within SFTSV-infected Vero E6 cells (MOI-0.01, MOI-0.1). The infected cells were divided into two groups: one group was subjected to fixation only (Figure 2A), whereas the other group underwent both fixation and membrane permeabilization (Figure 2B). As antibodies generally cannot penetrate the cell membrane, it is assumed that in the non-permeabilized group, antibodies recognize only the antigens present on the cell membrane surface. For [^111^In]In-DTPA-cIgG, no significant differences in the binding rates were observed between uninfected and SFTSV-infected cells in either the fixation-only group (Figure 2A) or the fixation and membrane-permeabilized groups (Figure 2B). In contrast, experiments with [^111^In]In-DTPA-N-mAb showed a significant increase in antibodies binding to cells as the infection level increased, with markedly higher binding compared with uninfected cells. Although the binding rate of [^111^In]In-DTPA-N-mAb to uninfected cells was similar to that of [^111^In]In-DTPA-cIgG, it was significantly higher in SFTSV-infected cells. This indicated that [^111^In]In-DTPA-N-mAb specifically bound to SFTSV-infected cells. Moreover, the binding rate in the membrane-permeabilized group (Figure 2B) was higher than that in the fixation-only group (Figure 2A). However, even without permeabilization, [^111^In]In-DTPA-N-mAb showed a significant increase in binding to infected cells. This suggests that [^111^In]In-DTPA-N-mAb specifically recognizes the N protein exposed on the surface of the infected cells. These findings suggest that the N protein in SFTSV is not restricted to the intracellular space but is also exposed on the cell membrane. Therefore, [^111^In]In-DTPA-N-mAb can bind to the N protein on the cell membrane in vivo, supporting its potential use as an in vivo imaging agent for SFTSV infection.

### 2.4. In Vivo Studies of [^111^In]In-DTPA-N-mAb in SFTSV- and Mock-Infected Mice

**Figure 3:**
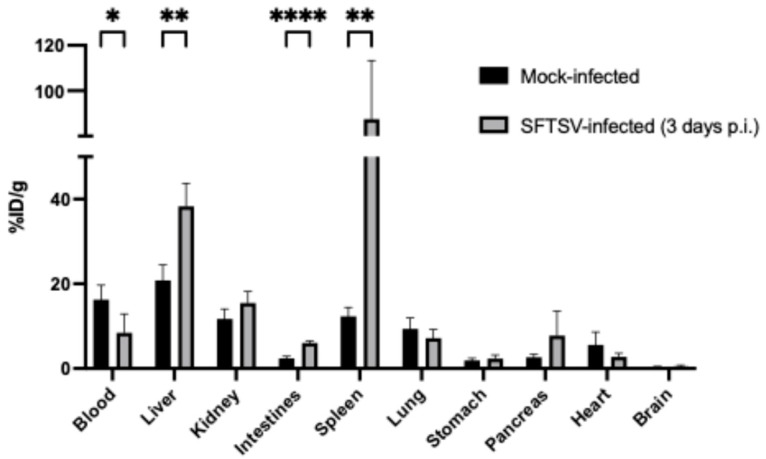
Biodistribution of [^111^In]In-DTPA-N-mAb in mock- or SFTSV-infected (3 days p.i.) A129 mice. [^111^In]In-DTPA-N-mAb was injected intravenously via the tail vein into the mock- or SFTSV- infected mice. To evaluate the biodistribution, after 24 h the mice were sacrificed, and the organs were dissected. * *p* < 0.05, ** *p* < 0.01, **** *p* < 0.0001 for mock-infected mice vs. SFTSV-infected mice (multiple unpaired *t*-test). Data are represented as the percentage of injected dose (%ID)/g ± SD (n = 5–6).

**Figure 4:**
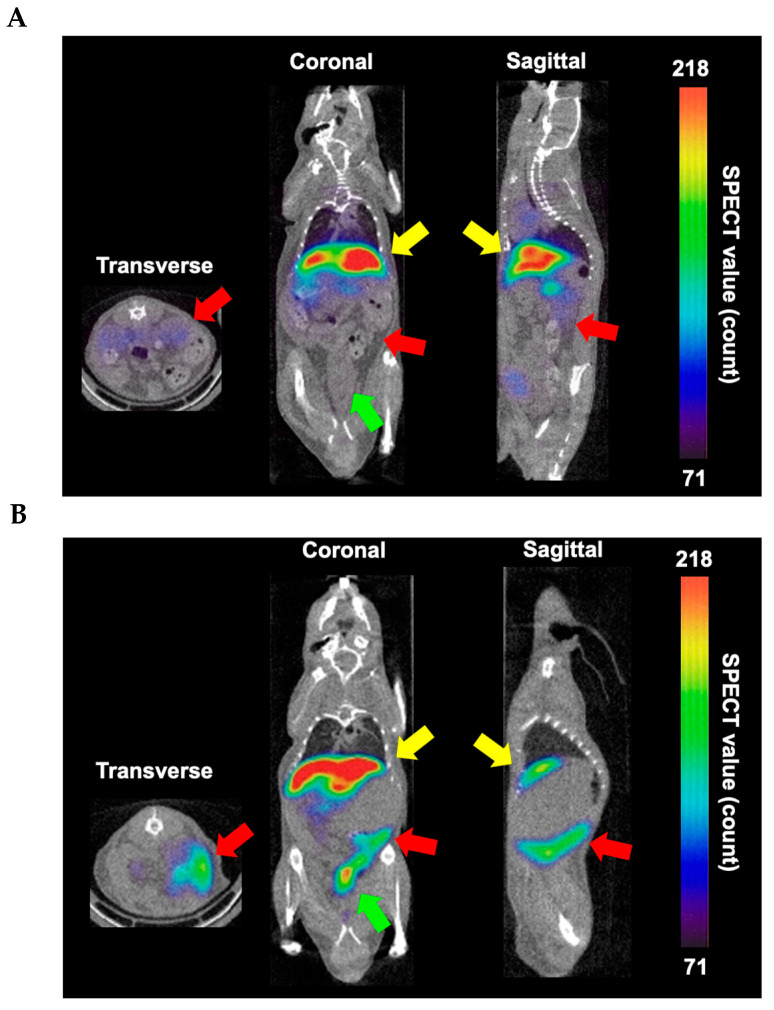
Representative axial (left panels), coronal (middle panels), and sagittal (right panels) SPECT/CT images of mock-infected (**A**) and SFTSV-infected (**B**) A129 mice 3 days after infection. SPECT/CT images were acquired 24 h after intravenous injection of [^111^In]In-DTPA-N-mAb. The arrows indicate the liver (yellow), spleen (red), and gastrointestinal tract (green).

**Figure 5:**
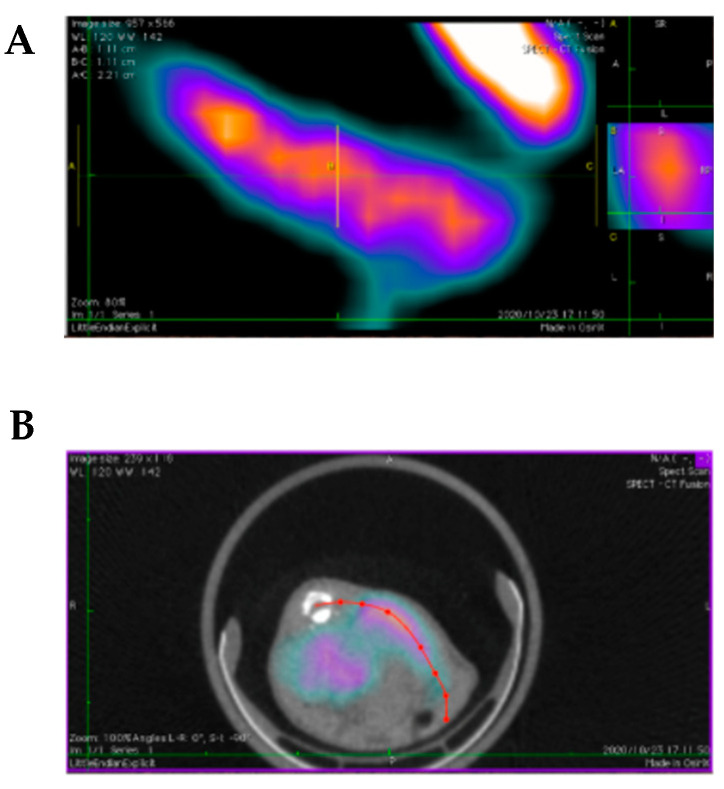
Analysis of the SPECT/CT images of [^111^In]In-DTPA-N-mAb in SFTSV-infected mouse using the CPR method. The cross-sectional image (**A**) was reconstructed along the red line in the SFTSV-infected mouse SPECT/CT imaging data (**B**).

Type I interferon receptor knockout (A129) mice are effective models for studying SFTSV pathogenesis in vivo. In previous studies, pathological changes were observed three days post-infection, just before the onset of mortality on day four [33,34]. Therefore, we used SFTSV-infected A129 mice at three days post-infection as a model for severe SFTS. Previous research has reported that radiolabeled antibodies exhibit high blood retention for several days [35]. Considering the long blood retention of antibodies, we administered [^111^In]In-DTPA-N-mAb intravenously via the tail vein to non-infected and SFTSV-infected mice and assessed its in vivo distribution at 24 h post-injection. Accumulation of [^111^In]In-DTPA-N-mAb in each organ is shown in Figure 3, and the detailed accumulation data are presented in Table S1. Blood accumulation was significantly lower in the SFTSV-infected mice than in the uninfected mice. In contrast, a significantly higher accumulation was observed in the liver, spleen, and intestines (Figure 3). Biodistribution of [^111^In]In-DTPA-cIgG in SFTSV-infected mice confirmed lower accumulation in these organs compared to [^111^In]In-DTPA-N-mAb (Figure S3), reinforcing the hypothesis that [^111^In]In-DTPA-N-mAb binds specifically to SFTSV. Similar evaluations were conducted using SPECT/CT to validate the tracer as an in vivo imaging agent for the SFTSV infection. [^111^In]In-DTPA-N-mAb was administered to both non-infected and 3-day post-infection SFTSV-infected mice, and SPECT/CT imaging was performed 24 h post-injection. Consistent with the in vivo experiments, accumulation in the liver was prominent in non-infected mice, with no detectable accumulation in the spleen (Figure 4A). In contrast, high accumulation was observed in the liver, spleen, and intestines of SFTSV-infected mice (Figure 4B). These results demonstrate that [^111^In]In-DTPA-N-mAb successfully visualized SFTSV-infected organs in mice 3 days post-infection, confirming its potential as a SPECT imaging agent. In the imaging data of Figure 4B, the region with high accumulation, indicated by a red arrow, presumably corresponding to the spleen, was unclear in the CT cross-sectional images. Therefore, using the acquired imaging data, we applied curved planar reconstruction (CPR) to create three-dimensional images to clarify the shapes of the accumulated organs (Figure 5A,B). The area of high accumulation of [^111^In]In-DTPA-N-mAb, indicated by the red arrow in Figure 4, was confirmed to be the spleen.

Indeed, while more biologically stable complexes such as DOTA [36] and CHX-DTPA [37] have been widely reported, [^111^In]In-DTPA is also considered a highly stable complex in vivo and continues to be extensively used in basic research [38]. To assess the potential stability of [^111^In]In-DTPA-N-mAb, a SPECT imaging study was carried out using [^111^In]InCl_3_, which is assumed to exhibit a biodistribution pattern similar to that of ^111^In dissociated from DTPA. As shown in Figure S4, [^111^In]InCl_3_ displayed minimal accumulation in tissues other than the kidneys 24 h after administration. In contrast, [^111^In]In-DTPA-N-mAb demonstrated high blood retention at the same time point, with limited accumulation in the kidneys (Figure 4B). These findings suggest that [^111^In]In-DTPA-NmAb may be stable in vivo.

It has been reported that in SFTSV-infected mice, viral RNA load increases in most organs, with particularly high levels in the spleen [33]. Additionally, the N protein is abundantly present in the lymph nodes and spleen of infected mice and is detected in the liver and kidneys [39,40]. In humans, SFTSV targets B cells, suggesting that the virus primarily affects the lymph nodes and spleen [41]. The results of this study align with these clinical findings, showing significantly higher accumulation of radioactivity in the liver and spleen of infected mice in an infection-dependent manner than in non-infected mice. Furthermore, [^111^In]In-DTPA-N-mAb showed significantly higher accumulation in the gastrointestinal tract, similar to the notable accumulation observed using [^18^F]FDG and [^68^Ga]Ga-citrate in PET imaging, compared to non-infected mice. In this study, splenomegaly was observed in the spleens of SFTSV-infected mice, along with stomach distension and liquefaction of its contents, consistent with previous reports [24]. Gastrointestinal symptoms such as vomiting and diarrhea are commonly reported in patients with SFTSV [42]. The significantly increased accumulation of [^111^In]In-DTPA-N-mAb in the gastrointestinal tract, lymph nodes, spleen, liver, and kidneys, which are considered target tissues of SFTSV, suggests that [^111^In]In-DTPA-N-mAb may specifically recognize SFTSV in clinical settings. The in vivo radioactivity distribution showed that [^111^In]In-DTPA-N-mAb accumulated significantly in organs that highly expressed the target N protein, correlating well with the in vitro cellular evaluations. This suggests that in SFTSV-infected mice, the N protein is exposed on the cell membranes of the infected tissues. In previous studies, no accumulation outside the intestinal system was observed for [^18^F]FDG or [^68^Ga]Ga-citrate, whereas [^111^In]In-DTPA-N-mAb demonstrated high accumulation in the spleen, an SFTSV target organ. This indicates that, unlike conventional imaging agents that detect inflammation, [^111^In]In-DTPA-N-mAb accurately reflects the localization of SFTSV. Compared to PCR and ELISA, which demonstrate exceptional sensitivity and specificity for SFTSV [43], SPECT with [^111^In]In-DTPA-N-mAb offers distinct advantages, including the ability to visualize SFTSV dynamics in infection model animals and identify residual viral distribution post-treatment. These features highlight its potential clinical value despite inherent limitations in sensitivity and specificity. In vivo studies in mice revealed nonspecific accumulation of the antibody in the liver and spleen. To mitigate this issue, strategies such as antibody fragmentation or PEGylation could be considered. For clinical applications, the deep tissue localization of the virus might pose challenges for imaging feasibility. Developing peptide-based agents capable of penetrating deeper tissues may help address this limitation. Furthermore, exploring compounds of various sizes could optimize SFTSV imaging probes. Overall, [^111^In]In-DTPA-N-mAb is expected to be developed as a tool for elucidating disease dynamics using SFTSV-infected animal models, as well as for non-invasive imaging diagnostics to determine the SFTSV infection status in patients.

## 3. Materials and Methods

### 3.1. Materials

Unless otherwise specified, commercially available first- or special-grade reagents were used in all the experiments. Anti-SFTSV monoclonal antibodies (N4A10, GN2D4, and GC3B4) against the Gn, Gc, and N proteins, which were expressed by hybridomas constructed using the spleens of SFTSV-infected mice, were provided by the Department of Virology, Institute of Tropical Medicine, Nagasaki University. Mouse IgG Isotype Control (cIgG) was purchased from Southern Biotechnology Associates, Inc. (Birmingham, AL, USA), and S-2-(4-isothiocyanatobenzyl)-diethylenetriamine pentaacetic acid (p-SCN-Bn-DTPA) was obtained from Macrocyclics Inc. (Dallas, TX, USA). For solvent exchange and purification of the samples, Amicon Ultra 0.5 mL Ultracel (molecular weight cutoff: 30 kDa, 100 kDa) from Merck (Darmstadt, Germany) was used. [^111^In]InCl_3_ was purchased from Nihon Medi Physics Co. Ltd. (Tokyo, Japan). The radioactivity of each sample was measured using an Autowell γ-counter (ARC-7010B; ALOKA Co., Ltd., Tokyo, Japan). For matrix-assisted laser desorption ionization time-of-flight (MALDI-TOF) mass spectrometry, we used an Ultraflex TOF/TOF (Bruker Daltonics, Bremen, Germany). The sample target used was the Anchorchip™ var/384 TF (Bruker Daltonics).

The mice used in the experiments included 5-week-old male ddY mice (weighing 24–26 g) purchased from Japan SLC, Inc. (Shizuoka, Japan), as well as IFNARKO (interferon-α/β receptor knockout, A129) mice purchased from B&K Universal Ltd. (North Humberside, UK). The latter were bred in the facilities at Nagasaki University, Nagasaki, Japan.

### 3.2. Preparation of SFTS Virus (SFTSV)-Infected Cells

Vero E6 cells (10^4^ × 10^5^ cells/well, 300 µL/well) were seeded into Millicell EZ slides (Millipore, Molsheim, France) and incubated at 37 °C in 5% CO_2_ for 24 h. The cells were then infected with SFTSV (strain NB-13) at MOI-0.01 and MOI-0.1, with 300 µL of inoculum per well, and incubated at 37 °C in 5% CO_2_ for 36 h. After infection, 200 µL/well of 4% paraformaldehyde was added, and the cells were fixed at 37 °C for 30 min. Subsequently, 200 µL/well of 1% NP-40 was added, and the cells were permeabilized at 37 °C for 30 min. A separate group of cells was prepared by performing the fixation step without permeabilization.

### 3.3. Immunofluorescence Staining of SFTSV-Infected Cells

SFTSV-infected or mock-infected cells were blocked with 5 *w*/*v*% Block Ace (KAC Co., Ltd., Kyoto, Japan) at room temperature for 1 h. Anti-mouse SFTSV monoclonal antibodies (N-mAb, Gn-mAb, and Gc-mAb) were used as primary antibodies, added at concentrations of 5 µg/mL, and incubated at 37 °C in 5% CO_2_ for 1 h. FITC-conjugated anti-mouse IgG (1:500) was then added as the secondary antibody and incubated under the same conditions for 1 h. A small amount of DAPI, a nuclear stain, was subsequently added to the plates, and the cells were observed under a fluorescence microscope BZ-X710 (KEYENCE, Osaka, Japan).

### 3.4. Synthesis of DTPA-Antibodies

Anti-SFTSV antibody (N-mAb) or mouse IgG Isotype Control (2.5 mg/mL) and p-SCN-Bn-DTPA (0.5 mg/mL) were mixed in a 0.2 M NaHCO_3_ solution and shaken at 37 °C for 1 h. After shaking, the reaction mixture was purified using an Amicon Ultra 0.5 mL Ultracel 100 kDa (Merck Millipore, Billerica, MA, USA).

### 3.5. Radiosynthesis of [^111^In]In-DTPA Antibodies

To a 0.67 mL solution of DTPA-cIgG or DTPA-N-mAb (150 µg) in 0.1 M acetate buffer (pH 6.0), 1.0 mL (74 MBq) of [^111^In]InCl_3_ solution was added and left to stand at 37 °C for 30 min. Purification was performed by centrifugation at 14,000× *g* for 10 min using an Amicon Ultra-0.5 mL Ultracel 100 kDa filter. The product was recovered by inverting the column and centrifuging at 1000× *g* for 2 min. Radioactivity before and after the reaction using a γ-counter was measured to determine the radiochemical yield. Radiochemical purities of ^111^In-labeled antibodies were determined by glass microfiber chromatography paper impregnated with silica gel (iTLC-SG, Agilent Technologies, CA, USA) developed with 0.15 M sodium citrate.

### 3.6. Cellular Binding of [^11I^In]In-DTPA Antibody in SFTS-Infected Cells

The cellular binding study was performed according to the previous study [44,45]. Fixed cells were prepared using only paraformaldehyde, while fixed and permeabilized cells were prepared using both paraformaldehyde and NP-40, as in the fluorescence staining experiments. [^111^In]In-DTPA antibodies ([^111^In]In-DTPA-cIgG, [^111^In]In-DTPA-N-mAb) at the 6.7–41 kBq dose level were added to SFTSV-infected cells (MOI-0.01, MOI-0.1) or non-infected cells and incubated at 37 °C for 120 min. The cells were washed twice with cold heparin (20 units/mL PBS) and lysed using Cell Lysis Buffer M (FUJIFILM Wako Pure Chemical Corporation, Osaka, Japan). After collecting the cells, radioactivity was measured using a γ-counter, and protein concentration was measured by the Bradford method. Cellular uptake is expressed as the applied dose (AD) per milligram of protein (%AD/mg protein).

### 3.7. Biodistribution of ^111^In-DTPA-N-mAb in SFTSV-Infected Mice

Animal experiments were conducted in accordance with our institutional guidelines and were approved by the Nagasaki University Animal Care Committee. SFTSV-infected mice were prepared by subcutaneously inoculating A129 mice (6 weeks old, female) with 1 × 10^6^ ffu SFTSV in an EMEM solution containing 2% FBS. SFTSV-infected mice were used for experiments 3 days after infection. Similarly, non-infected control mice were prepared by subcutaneously inoculating A129 mice with EMEM solution containing 2% FBS. Each mouse was intravenously administered 100 µL (40 kBq) of [^111^In]In-DTPA-N-mAb via the tail vein. After 24 h, the mice were euthanized, blood was collected, and the major organs were excised. The samples were weighed and the radioactivity in each tissue sample was represented as a percentage of the injected dose per gram of tissue (%ID/g).

### 3.8. Small-Animal SPECT/CT Imaging of SFTSV-Infected Mice

SPECT/CT imaging of SFTSV- and mock-infected mice was conducted using the Triumph combined PET/SPECT/CT system (TriFoil Imaging Inc., Northridge, CA, USA). Each mouse received a tail vein injection of [^111^In]In-DTPA-N-mAb (5.8–7.2 MBq) or [^111^In]InCl_3_ (25.7 MBq). Immediately after the injection, the mice were anesthetized with 1.5% (*v*/*v*) isoflurane. SPECT imaging was performed using a four-head γ-camera equipped with single pinhole collimators (1.0 mm diameter, 90 mm focal length). SPECT data acquisition lasted for 33 min (rotation radius 50 mm, rotation angle 360°, 64 projections, 40 s per projection), beginning 24 h after intravenous injection. CT imaging was performed with X-ray acquisition settings of 60 kV and 128 projections while the animals were kept in the same position. SPECT data were reconstructed using the 3D-Maximum-Likelihood Expectation Maximization (3D-MLEM) algorithm with 50 iterations, and SPECT/CT images were processed using OsiriX MD software (Pixmeo, Geneva, Switzerland) and AMIDE Imaging Software (version 1.0.4).

## 4. Conclusions

This study demonstrated that [^111^In]In-DTPA-N-mAb is a promising in vivo imaging agent for detecting an SFTSV infection. The antibody effectively targeted the N protein, resulting in substantial accumulation in infected tissues, including the spleen and intestine. Imaging results aligned with the in vitro data, underscoring the potential of [^111^In]In-DTPA-N-mAb to noninvasively visualize the dynamics of SFTSV infection. These findings support its use in clinical settings for the diagnosis and monitoring of SFTS. Future advancements with this imaging agent may deepen our understanding of the pathogenesis of SFTSV and enhance patient management.

## Data Availability

The original contributions presented in the study are included in the article/Supplementary Materials. Further inquiries can be directed to the corresponding author.

## Supplementary Materials

The following supporting information can be downloaded at: https://www.mdpi.com/article/10.3390/molecules30010038/s1, Table S1: Biodistribution of radioactivity of [^111^In]In-DTPA-N-mAb in mock- or SFTSV-infected A129 mice. Figure S1: MALDI-TOF MS spectrum of N-mAb (A) and DTPA-N-mAb (B). Figure S2: Quantified values of autoradiography of iTLC for [^111^In]In-DTPA-N-mAb (A) and [^111^In]In-DTPA-cIgG (B) developed with 0.15 M sodium citrate. Figure S3: Biodistribution of [^111^In]In-DTPA-cIgG in SFTSV-infected (3 days p.i.) A129 mice. [^111^In]In-DTPA-cIgG was injected intravenously via the tail vein into the SFTSV infected mice. To evaluate the biodistribution, after 24 h the mice were sacrificed, and the organs were dissected. Data are represented as the percentage of injected dose (%ID)/g ± SD (n = 4). Figure S4: Representative axial (left panels), coronal (middle panels), and sagittal (right panels) SPECT/CT images of a normal ddY mouse acquired 24 h after intravenous injection of [^111^In]InCl_3_. The arrows indicate the kidneys (magenta).
