# Supplementary material for: Development of 111In-Labeled Monoclonal Antibodies Targeting SFTSV Structural Proteins for Molecular Imaging of SFTS Infectious Diseases by SPECT
Source: Molecules. 2024 Dec 26;30(1):38. doi: 10.3390/molecules30010038 (PMC11721709; doi:10.3390/molecules30010038)
Supplement: Supplementary file 1 [file molecules-30-00038-s001.zip › molecules-3333941-supplementary.pdf]

Supporting Information

Development of ^{111}In-labeled monoclonal antibodies targeting SFTSV structural proteins for molecular imaging of SFTS infectious diseases by SPECT

Takeshi Fuchigami[1]*, Mya Myat Ngwe Tun[2,3], Yusuke Tanahara[4], Kodai Nishi[5], Sakura Yoshida[4], Kazuma Ogawa[1,6], Morio Nakayama[4], Daisuke Hayasaka[7]*

1. Laboratory of Clinical Analytical Sciences, Graduate School of Medical Sciences, Kanazawa University, Kakuma-machi, Kanazawa, Ishikawa 920-1192, Japan;

2. Center for Vaccines and Therapeutic Antibodies for Emerging Infectious Diseases, Shimane University, Izumo 690-8504, Japan;

3. Department of Virology, Department of Tropical Viral Vaccine Development, Institute of Tropical Medicine, Nagasaki University, Nagasaki 852-8523, Japan;

4. Department of Hygienic Chemistry, Graduate School of Biomedical Sciences, Nagasaki University, 1-14 Bunkyo-machi, Nagasaki 852-8521, Japan;

5. Department of Radioisotope Medicine, Atomic Bomb Disease Institute, Nagasaki University, 1-12-4 Sakamoto, Nagasaki 852-8523, Japan;

6. Institute for Frontier Science Initiative, Kanazawa University, Kakuma-machi, Kanazawa, Ishikawa 920-1192, Japan;

7. Laboratory of Veterinary Microbiology, Joint Graduate School of Veterinary Medicine, Yamaguchi University, 1677-1 Yoshida, Yamaguchi 753-8511, Japan;

Corresponding Authors

Takeshi Fuchigami − Laboratory of Clinical Analytical Sciences, Graduate School of Medical Sciences, Kanazawa University, Kakuma-machi, Kanazawa, Ishikawa 920-1192, Japan, Kakuma-machi, Kanazawa, Ishikawa 920-1192, Japan;

orcid.org/0000-0001-8141-1212; Email: t-fuchi@p.kanazawa-u.ac.jp*

Daisuke Hayasaka − Laboratory of Veterinary Microbiology, Joint Graduate School of Veterinary Medicine, Yamaguchi University, 1677-1 Yoshida, Yamaguchi 753-8511, Japan; Email: dhaya@yamaguchi-u.ac.jp*

Table of Contents

Figure S1 MALDI-TOF MS spectrum of antibodies ········· S4

Figure S2 Characterization of ^{111}In-labeled antibodies via iTLC ········· S5

Figure S3 Biodistribution of [^{111}In]In-DTPA-cIgG in SFTSV-infected mice ········· S6

Figure S4 SPECT/CT images of [^{111}In]$InCl_3$ in normal mice ········· S7

Table S1 Detailed biodistribution data of [^{111}In]In-DTPA-N-mAb in mice ········· S8

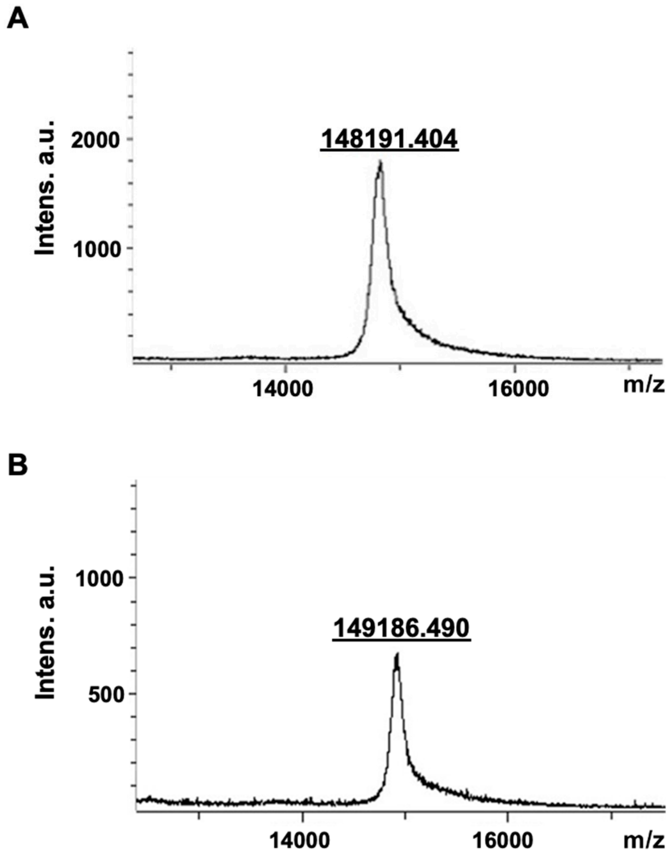

Figure S1. MALDI-TOF MS spectrum of N-mAb (A) and DTPA-N-mAb (B).

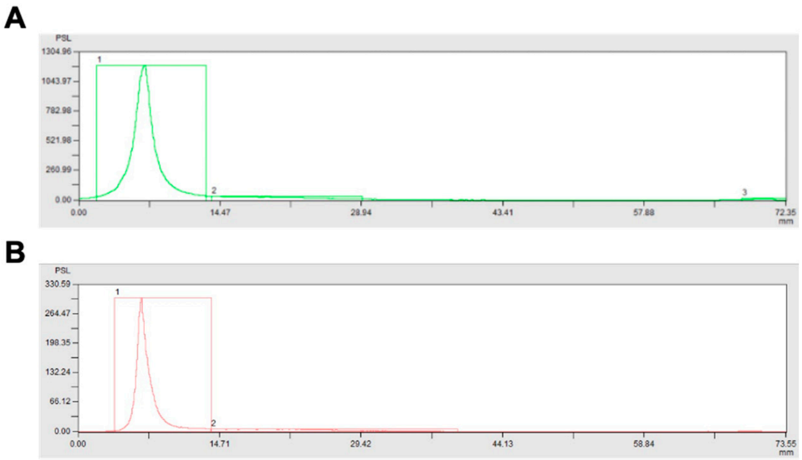

Figure S2. Quantified values of autoradiography of iTLC for [^{111}In]In-DTPA-N-mAb (A) and [^{111}In]In-DTPA-cIgG (B) developed with 0.15 M sodium citrate.

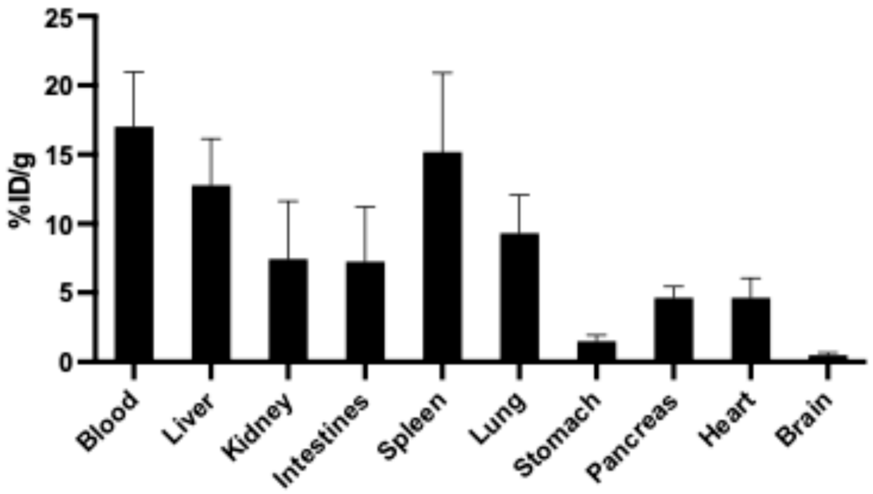

Figure S3. Biodistribution of [^{111}In]In-DTPA-cIgG in SFTSV-infected (3 days p.i.) A129 mice. [^{111}In]In-DTPA-cIgG was injected intravenously via the tail vein into the SFTSV infected mice. To evaluate the biodistribution, after 24 h the mice were sacrificed, and the organs were dissected. Data are represented as the percentage of injected dose (%ID)/g ± SD (n = 4).

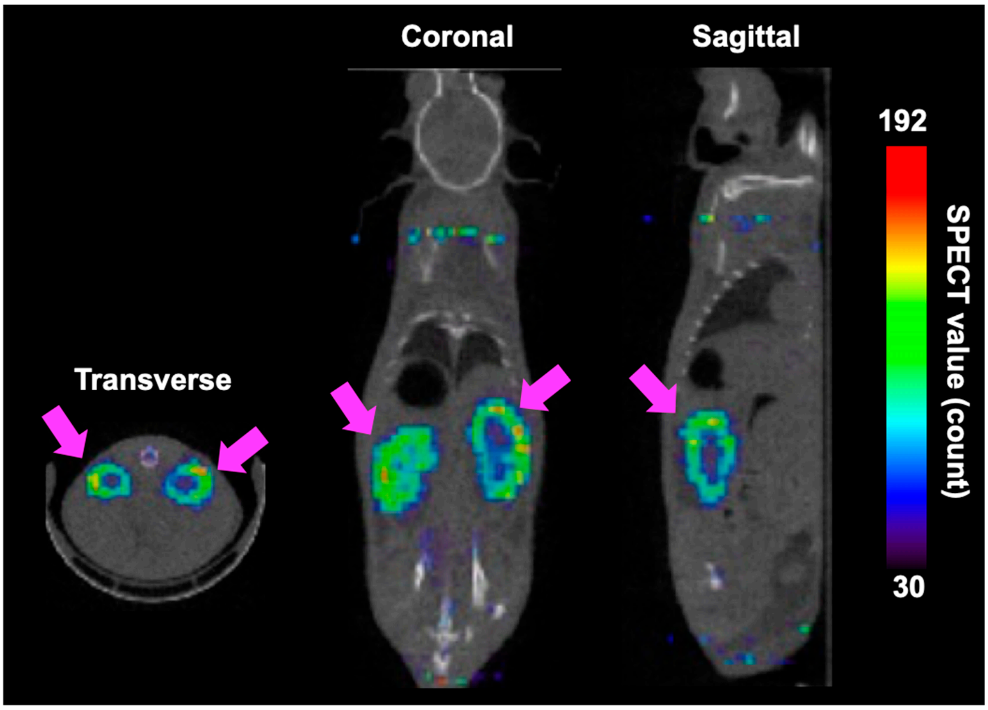

Figure S4. Representative axial (left panels), coronal (middle panels), and sagittal (right panels) SPECT/CT images of a normal ddY mouse acquired 24 h after intravenous injection of [^{111}In]$InCl_3$. The arrows indicate the kidneys (magenta).

Table S1. Biodistribution of radioactivity of [^{111}In]In-DTPA-N-mAb in mock- or SFTSV-infected A129 mice.

Tissues	Mock-infected	SFTS-infected (3 days p.i.)
Blood	16.25 (3.43)	8.42 (4.48)
Liver	20.80 (3.66)	38.28 (5.45)
Kidney	11.70 (2.37)	15.44 (2.75)
Intestines	2.37 (0.51)	6.02 (0.46)
Spleen	12.33 (2.09)	87.48 (25.82)
Lung	9.37 (2.56)	7.14 (2.07)
Stomach	1.90 (0.50)	2.30 (0.85)
Pancreas	2.65 (0.70)	7.74 (5.79)
Heart	5.55 (3.04)	2.76 (0.84)
Brain	0.42 (0.08)	0.56 (0.19)

Expressed as % injected dose per gram. Each value represents the mean (SD) for three or four animals.
